# Evaluating Bioinformatics Processing of Somatic Variant Detection in cfDNA Using Targeted Sequencing with UMIs

**DOI:** 10.3390/ijms252111439

**Published:** 2024-10-24

**Authors:** Yixin Lin, Mads Heilskov Rasmussen, Mikkel Hovden Christensen, Amanda Frydendahl, Lasse Maretty, Claus Lindbjerg Andersen, Søren Besenbacher

**Affiliations:** 1 Department of Molecular Medicine, Aarhus University Hospital, 8200 Aarhus, Denmark; yixinlin@clin.au.dk (Y.L.); cla@clin.au.dk (C.L.A.); 2 Department of Clinical Medicine, Aarhus University, 8000 Aarhus, Denmark; 3 Bioinformatics Research Centre, Department of Molecular Biology and Genetics, Aarhus University, 8000 Aarhus, Denmark

**Keywords:** cell-free DNA, low-frequency variant calling, UMI sequencing, benchmarking, cancer sample classification

## Abstract

Circulating tumor DNA (ctDNA) is a promising cancer biomarker, but accurately detecting tumor mutations in cell-free DNA (cfDNA) is challenging due to their low frequency and sequencing errors. Our study benchmarked Mutect2, VarScan2, shearwater, and DREAMS-vc using deep targeted sequencing of cfDNA with Unique Molecular Identifiers (UMIs) from 111 colorectal cancer patients. Performance was assessed at both the mutation level (distinguish tumor variants from errors) and the sample level (detect if an individual has cancer). Additionally, we investigated the effects of various UMI grouping and consensus strategies. The shearwater-AND variant calling method demonstrated the highest precision in detecting tumor-derived mutations from plasma, and reached the highest ROC-AUC of 0.984 for sample classification in tumor-informed cfDNA analyses. DREAMS-vc exhibited the highest ROC-AUC of 0.808 for sample classification in tumor-agnostic studies. We also found that sequencing depth differences in PBMCs could lead to false positives, particularly with VarScan2 and Mutect2, which was addressed by downsampling to equivalent mean depths. Additionally, network-based UMI grouping methods outperformed those using identical UMIs when all reads were retained. Our findings emphasize that the optimal variant caller depends on the study context—whether focused on mutation or sample classification, and whether conducted under tumor-informed or tumor-agnostic conditions.

## 1. Introduction

Although a traditional tissue biopsy enables accurate prognosis of cancer status and provides molecular targets for precision medicine, it is restricted by the invasive acquisition of solid tissues and cannot reflect the real-time status of tumor progression by repeated examination [1,2,3]. Therefore, many studies have investigated new biomarkers for noninvasive cancer screening and monitoring. CfDNA, i.e., DNA fragments released from cells to body fluids, was first detected in plasma by Mandel and Metais in 1948 [4]. In cancer patients, a fraction of their cfDNA will consist of circulating tumor DNA (ctDNA) shed by tumor cells into blood, urine, and other peripheral body circulating fluids [5]. Some of these tumor DNA fragments will contain the mutational characteristics (e.g., single nucleotide variants (SNVs), indels, etc.) of the tumor, making them distinguishable from cfDNA fragments stemming from healthy cells [5,6]. The half-life of cfDNA is short [7], and the tumor fraction of the cfDNA is correlated to the size and stage of the tumor [8,9]. These characteristics make ctDNA a promising real-time marker of tumor burden and a promising approach for early-stage tumor prognosis, monitoring therapy response, and in-time relapse detection [5,7,10].

The advancement of next-generation sequencing has provided good tools for detecting somatic mutations. However, the low frequency of ctDNA in blood (often less than 0.1% in patients with a low tumor burden) is a challenge for sensitive detection [11,12]. The deep targeted sequencing of either fixed or bespoke panels is an approach designed to improve the chances of detecting somatic mutations in cfDNA [3]. DNA damage and polymerase-mediated nucleotide misincorporations can, however, cause sequencing errors at rates as high as 1% per base, which can confound variant calling dramatically [13]. The addition of UMIs before amplification can correct the majority of sequencing errors by indexing the DNA molecules with short unique tags made of arbitrary oligonucleotides [14,15]. By redundant sequencing, sequencing errors then can be mitigated through the assembly of multiple reads originating from the same DNA fragment [16]. Consequently, true somatic variants that exist in all reads within a UMI group (i.e., reads with an identical UMI sequence and genomic position) can be distinguished from errors that are only present in a proportion of the reads [14]. The original molecule can be recreated by creating a consensus read within the UMI group [17] by assigning the nucleotide with the maximum posterior probability base by base. Nevertheless, UMI-seq cannot remove all errors, as the UMI consensus calling cannot remove single-strand errors introduced before UMI ligation. Besides the DNA damage to the original molecule, wrong bases can also be inserted into the UMI tags after the first PCR cycle. To handle such errors in the UMI tags, specialized UMI-grouping methods that cluster nearly identical UMI tags have been created [18].

**Figure 1:**
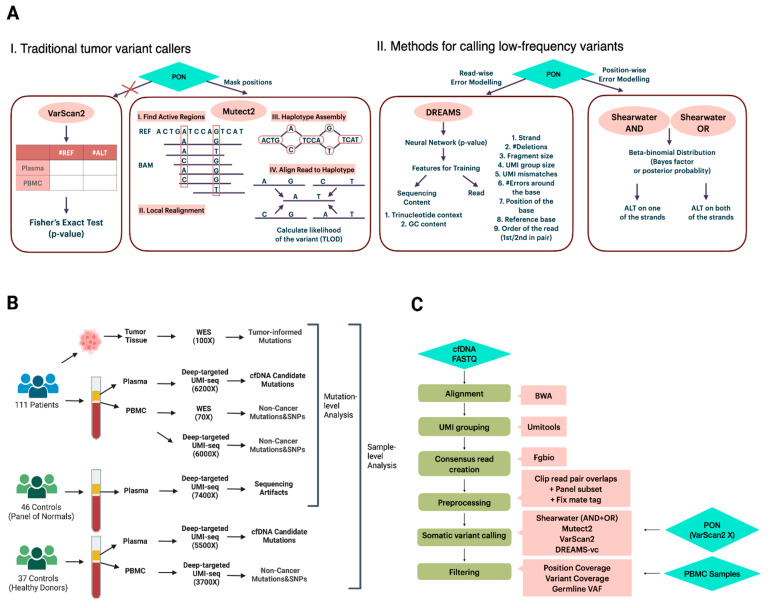
(**A**) Graphical illustration of the primary concept behind the variant callers VarScan2, Mutect2, DREAMS, and shearwater. (**B**) Study design and the features of all datasets. The depths of deep targeted UMI-seq data are the mean depth after UMI collapsing. (**C**) The main six steps of the bioinformatics pipeline. A more detailed pipeline version can be seen in Supplementary Figure S1. (Created with BioRender.com).

Unlike germline variants whose variant allele frequency (VAF) is distributed around 50% or 100%, downstream ctDNA somatic variant calling is a crucial step in need of precise modeling of error profiles. The performance of conventional state-of-the-art somatic variant calling methods is not well examined on cfDNA data. VarScan2 (Figure 1A), a well-known tumor variant caller, compares the signal differences in tumor-normal pairs by Fisher’s exact test and outputs the variants under a significant *p*-value [19]. Rather than evaluating signals per position, Mutect2 (Figure 1A), a haplotype-based variant caller, realigns reads within the active regions and aligns reads to the de Bruijn graph representing haplotypes to estimate a variant probability [13,20]. This realignment gives it an advantage in complex regions where reads might be incorrectly aligned [13]. In addition to variant calling methods that primarily focus on tumor mutations with VAFs exceeding 10%, there is a growing interest in approaches specifically designed for calling low-frequency variants from targeted sequencing data. Two such methods are the deepSNV [12] and shearwater (Figure 1A) [21] algorithms, both of which model the nucleotide counts of either a paired normal sample or a group of controls at each site using a beta-binomial distribution. This approach enables the estimation of position- and nucleotide-wise background error rates across samples. The difference between deepSNV and shearwater is the statistic used to score variants: deepSNV uses a likelihood ratio test, while shearwater uses a Bayes factor [12,21].

The wide application of deep targeted UMI sequencing has led to the development of some UMI-based variant callers [15,22,23,24,25]. Many of the callers are, however, restricted to specific sequencing data or need parameters for error modeling that are hard to obtain, which limits their general applicability. For instance, iDES is designed for error suppression of CAPP-seq data, which incorporates barcode hybridization of patient-specific hotspots and works by calling ctDNA-positive patients rather than mutations [24]. SmCounter2 [25] provides variant-calling only, but its specific filters are not effective on UMI consensus reads generated by the commonly used open-source bioinformatic UMI software fgbio [26]. DREAMS-vc (Deep Read-level Modeling of Sequencing-errors) [22] is a newly developed deep learning approach for detecting somatic ctDNA SNVs. Its error model is trained using a range of read and sequencing context features from control samples (Figure 1A), such as GC content, UMI group size, read position, and trinucleotide context [22]. By leveraging a neural network, DREAMS-vc is capable of estimating the error rate of each read position [22]. Consequently, it is recognized as a read-wise modeling method for low-frequency variant calling.

Given the critical role of detecting low-frequency mutations in liquid biopsy samples for cancer detection and the current lack of comparison regarding the performance among different variant callers, we report the results from a comprehensive and rigorous benchmarking of the callers VarScan2, Mutect2, shearwater, and DREAMS-vc in both mutation-level and sample-level scenarios. Furthermore, to gain insights into optimal UMI processing, we report results from a comparison of the UMI grouping methods from the UMI-tools software [18] and the probabilistic consensus methods from the fgbio bioinformatic suite [26]. The presented workflows and method evaluations will be useful in clinical oncology, and can help identify targetable mutations or detect patients with residual disease or relapse.

## 2. Results

### 2.1. Comparison of the Ability to Correctly Identify Tumor Mutations in cfDNA

To benchmark the accuracy of different variant callers (Figure 1A), we used deep targeted, UMI-based cfDNA data from pre-operational plasma from 111 colorectal cancer patients. The whole-exome sequencing (WES) of the primary tumors and paired peripheral blood mononuclear cells (PBMC) yielded a set of 209 robustly identified SNVs within the 15,369 bp targeted region (see Section 4 and Supplementary Table S1). A cfDNA call was considered a true positive mutation if it also was identified in the patient’s tumor, while all remaining SNV calls were deemed false positive mutations. Three of the four variant callers also made use of a Panel of Normals (PON) consisting of data from 47 healthy individuals to either train an error model or filter recurrent technical artifacts (Figure 1B).

**Figure 2:**
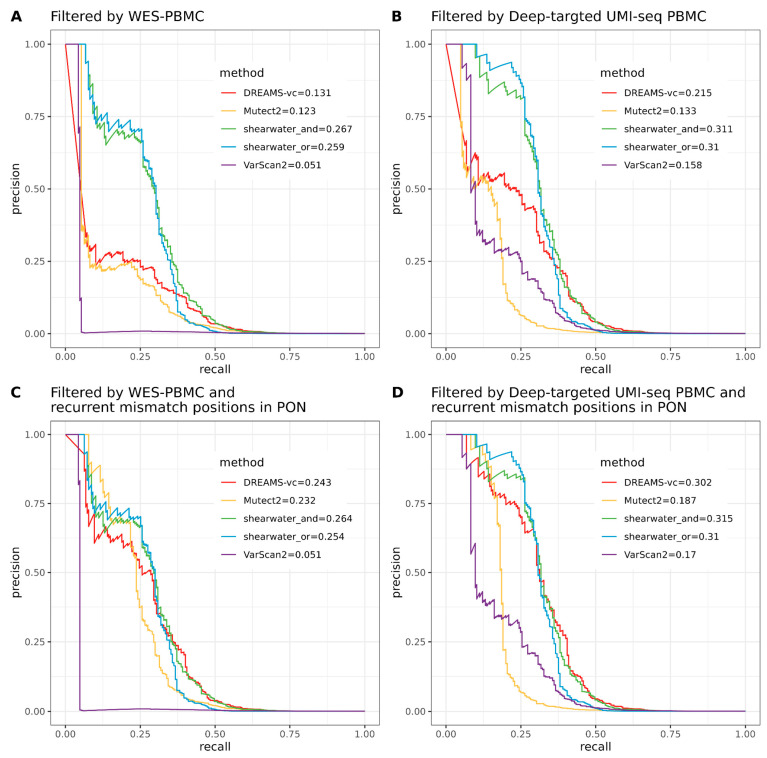
(**A**) Precision–recall curve (PR-curve) for mutation classification when using WES-seq PBMC to filter non-tumor mutations. (**B**) PR-curve for mutation classification when using deep targeted UMI-seq PBMC to filter non-tumor mutations. (**C**) PR-curve for mutation classification when using WES-seq PBMC to filter non-tumor mutations, with additional filtering of the problematic regions indicated by the PON, which have a larger than 1% mismatch rate and are detected in more than one normal sample. (**D**) PR-curve for mutation classification when using deep targeted UMI-seq PBMC to filter non-tumor mutations, with additional filtering of the problematic regions indicated by the PON, which have a larger than 1% mismatch rate and are detected in more than one normal sample.

In addition to the WES data from the PBMC, we also subjected the paired PBMC samples to deep targeted UMI-based sequencing to investigate the difference between high coverage (targeted) and low coverage (WES) normal control data for the filtering of non-tumor cfDNA variants. When using a WES PBMC for filtering, shearwater-AND exhibited the highest area under the precision–recall curve (PR-AUC), followed by Shearwater-OR, DREAMS-vc, Mutect2, and VarScan2 (Figure 2A). Filtering with a deep targeted UMI-seq PBMC significantly increased the PR-AUCs of all variant callers (Figure 2B), highlighting the benefits of deeper sequencing for eliminating variants not stemming from the tumor, such as clonal hematopoiesis of indeterminate potential (CHIP) mutations. Shearwater-AND and Shearwater-OR continued to demonstrate the best precision for calling mutations from cfDNA. Notably, Shearwater-OR achieved a precision higher than 0.9 when the recall was below 0.25, but was surpassed by shearwater-AND for higher recall values. This aligns with the underlying theories of shearwater, where Shearwater-OR requires both strands to contain the specific alternative, resulting in higher precision but reduced sensitivity.

All methods, except DREAMS-vc, achieved a precision of 1 at low recall rates, regardless of the sequencing strategy used for PBMC (Figure 2A,B). The challenge for DREAMS-vc was that among its most confident variant calls (*p*-value of 0), 65% (*n* = 26) and 35% (*n* = 7) were false positives when using WES PBMC and deep targeted UMI-seq PBMC for filtering, respectively. Of these, 24/26 and 6/7 were the same variant (chr4:152328179_G/C) called in different plasma samples. The average VAF of the variant was larger than 1% in both the PON and the plasma samples (1.2% for deep targeted UMI-seq PBMC, 1.4% for both PON and WES PBMC), indicating that this position has an unusually high error rate. Such high error rates might be challenging to account for using a model based on read and context features, like DREAMS-vc. However, they could potentially be detected by methods that utilize the PON to estimate position-specific error rates. When additional filtering was performed by excluding recurrent mismatch positions (see Section 4), the performance of DREAMS-vc and Mutect2—which do not include a per-position error rate estimation—significantly improved, with no false positives detected among the highest-quality variants (Figure 2C,D).

We expect that the main factor affecting the ability to call a variant correctly is its frequency in the blood. To test this, we split the putative variants in the dataset based on their VAF (number of reads containing the alternative allele) to evaluate how variant callers perform across a broad spectrum of allele frequencies. As shown in Supplementary Figure S2, none of the variant callers can reliably detect variants with a VAF below 1 × 10^−4^. For ctDNA mutations in the range of (1 × 10^−4^, 1 × 10^−3^], some variant callers can detect a few correctly, but all fail to call most variants correctly. When cfDNA mutations were within the range of (1 × 10^−3^, 1 × 10^−2^], all variant callers except Mutect2 achieved a PR-AUC above 0.5, and when the VAF was above 1%, all four variant callers got PR-AUC values close to 1.

### 2.2. Comparison of the Ability to Correctly Identify Individuals with Cancer Based on cfDNA Mutations

Detecting tumor variants in cfDNA is essential for determining the presence of tumor DNA, either to diagnose cancer or to detect minimal residual disease (MRD) after treatment. A tumor-informed analysis can be conducted by checking in the cfDNA sample for mutations identified from the tumor biopsy. However, in previously undiagnosed individuals, a tumor-agnostic analysis is necessary since no tumor information is available. To compare the effect of different variant callers on the ability to correctly distinguish individuals with ctDNA from patients without ctDNA in their plasma, we compared the sequence data from the 111 cancer patients to sequence data from 37 healthy donors and evaluated the ability to perform cancer classification in both a tumor-informed and tumor-agnostic analysis.

**Figure 3:**
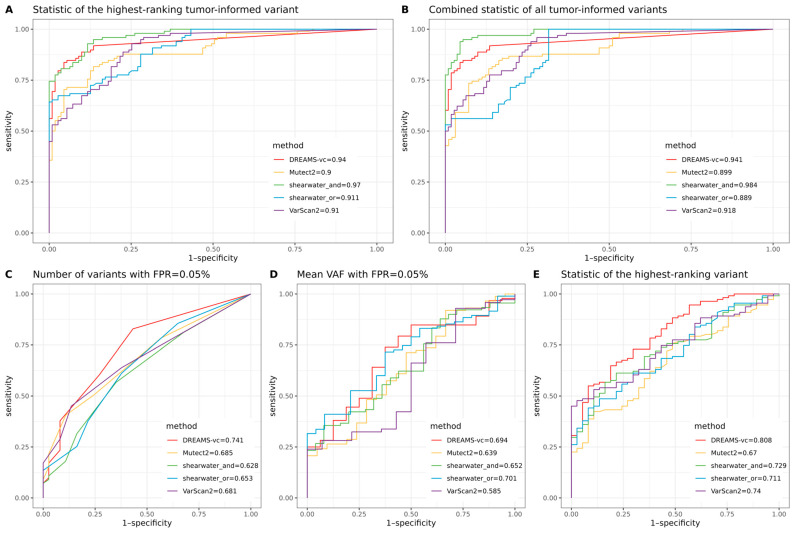
(**A**) ROC curve for sample classification in a tumor-informed study, based on the statistic of the highest-ranking tumor-informed variant. (**B**) ROC curve for sample classification in a tumor-informed study, based on the combined statistic of all the tumor-informed variants. (**C**) ROC curve for sample classification in a tumor-agnostic study, based on the number of variants called using a quality threshold estimated to have an FPR of 0.05%. (**D**) ROC curve for sample classification in a tumor-agnostic study, based on the mean VAF of variants called using a quality threshold estimated to have an FPR of 0.05%. (**E**) ROC curve for sample classification in a tumor-agnostic study, based on the statistic of the highest-ranking variant.

For the tumor-informed analysis, we assessed both the combined statistics of all corresponding tumor-informed mutations (see Section 4) and the statistic of the highest-ranking tumor-informed mutation in the plasma. “Statistic” here refers to the specific metrics used by the variant callers, including the *p*-value for VarScan2 and DREAMS-vc, TLOD for Mutect2, and posterior probability for shearwater. For the healthy donors who did not have cancer and consequently did not have a list of known tumor mutations, we created three different control data points by randomly pairing each control with the known tumor mutations of a case sample. The tumor-informed sample classification results reveal that shearwater-AND is the best variant caller for distinguishing patients with ctDNA from those without ctDNA in their plasma, achieving a ROC-AUC as high as 0.984 (Figure 3B), followed by DREAMS-vc with a ROC-AUC of 0.941 (Figure 3B). This ranking remains true regardless of whether the classification applies the statistic of the highest-ranking tumor-informed variant (Figure 3A) or the combined statistic of all the tumor-informed variants (Figure 3B).

Multiple criteria were selected to measure the ability to identify samples with ctDNA when there is no prior information on mutations. For the tumor-agnostic analysis, we evaluated three different criteria and compared their effects on classification. We assessed the number of called variants of each sample with a cutoff of 0.05% false positive rate (FPR), the mean variant allele frequency (VAF) of called variants of each sample with a cutoff of 0.05% FPR, and the statistic of the highest-ranking variant of each sample between cases and controls (see Section 4). The results become more complex when analyzed under tumor-agnostic sample classification. DREAMS-vc achieved the highest ROC-AUC (0.808) when using the highest-ranking variant for comparison (Figure 3E). Additionally, DREAMS-vc showed strong performance in measuring the number of called variants at a threshold of FPR = 0.05% and ranked second after Shearwater-OR in measuring the mean VAF of called variants at a threshold of FPR = 0.05%, with ROC-AUCs of 0.741 and 0.694, respectively (Figure 3C,D). Nonetheless, sample classification using variant calling alone remains challenging and requires further investigation.

### 2.3. Sequencing Depth as a Confounder in Sample Classification

**Figure 4:**
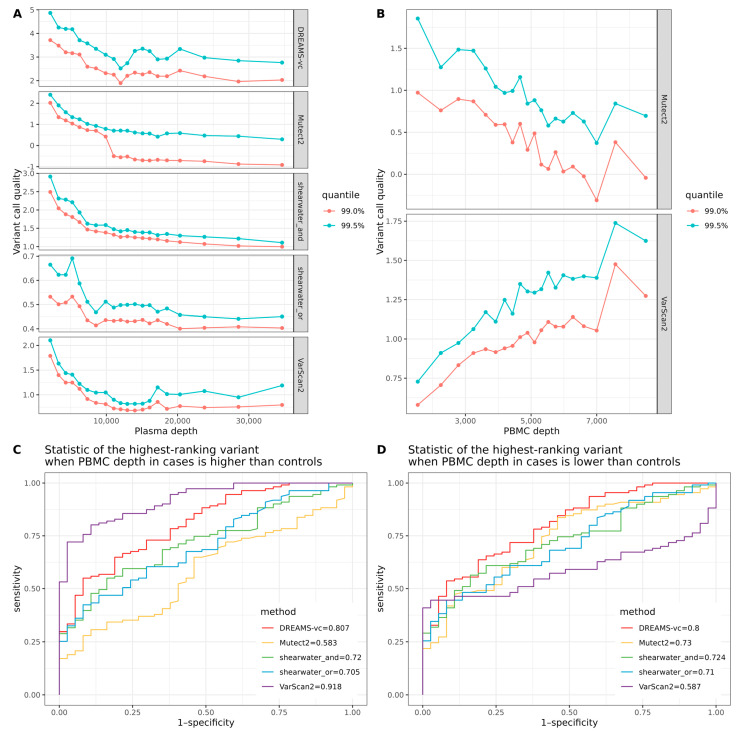
(**A**) The relationship between plasma depth and variant call quality. (**B**) The relationship between PBMC depth and variant call quality. (**C**) ROC curve for sample classification in tumor-agnostic study, based on the score of the highest-ranking variant. The analysis is based on the original data where PBMC depth (mean = 10,936X, sd = 5050) in cases is significantly higher than the controls (mean = 3710X, sd = 916). (**D**) ROC curve for sample classification in tumor-agnostic study, based on the score of the highest-ranking variant. The analysis is based on the downsampled data where PBMC depth in cases (mean = 2187X, sd = 1009) is significantly lower than the controls (mean = 3710X, sd = 916).

The quality of a variant call depends on the sequencing depth, and if the sequencing depth is correlated with the case–control status, it will be a confounder during sample classification [27]. To investigate the impact of sequence depth on the variant call quality (see Section 4), we grouped all the positions in the called regions into 20 bins based on the depth and calculated the 99% and 99.5% quantiles of the variant call qualities observed in the controls. Specifically, mutations with a higher variant call quality are more likely to be true tumor-derived mutations and are more likely to be called. The results shown in Figure 4A show that it is more likely to see significant false positives when the plasma depth is low. For the two variant callers that use normal controls in their calculation of the variant call qualities, different relationships between the number of extreme quality values and the depth of the PBMC were observed (Figure 4B). Varscan2 is more likely to report a high variant call quality if the depth in the PBMC is high, whereas Mutect2 is more likely to give a high quality to a variant call if the PBMC depth is low.

In the results presented in Figure 3, the PBMC sequence data of the cases were downsampled to have a mean depth similar to the controls. Had this downsampling not been performed, the ROC-AUC for VarScan2 reported in Figure 3E would have been 0.918 instead of 0.74 (Figure 4C). This would, however, only be an artifact of the differences in depth between cases and controls that would give false positive variants in the cases with higher quality scores due to their high PBMC depth. If the confounder was reversed and the PBMC depth in the cases was downsampled to half of that of the controls, the ROC-AUC for VarScan2 dropped to 0.587 (Figure 4D). The confounding effect of PBMC depth on Mutect2 was smaller, but still significant with the ROC-AUC going from 0.583 when the PBMC depth was higher in cases than controls to 0.73 when the PBMC depth was lower in cases than controls.

### 2.4. Comparing UMI Handling

The pipeline steps of UMI grouping and consensus collapsing are important as they can impact the number of false positives and the depth of tumor-derived mutations in the data awaiting for variant calling. Therefore, we explored various choices of UMI grouping by the UMI-tools software [18], such as “adjacency”, “cluster”, “directional”, “percentile”, and “unique”, and the minimum number of reads (e.g., 1 to 8, 10, and 15) within a group during UMI consensus by the fgbio suite. We tested the performance of different UMI handling strategies in combination with one particular variant caller. Because of good performance in terms of both precision and sensitivity, shearwater-AND was chosen to test the different UMI options.

**Figure 5:**
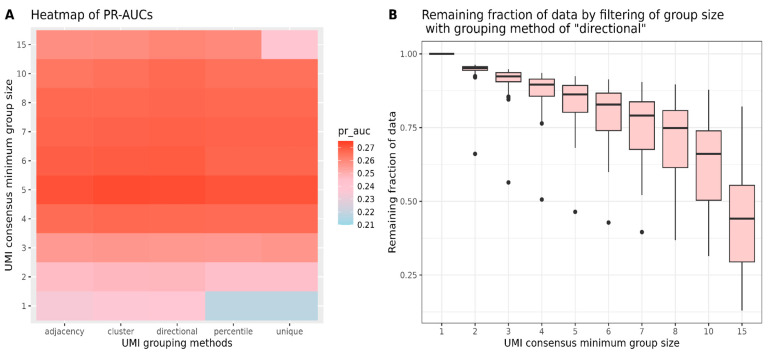
Different combinations of five UMI grouping methods (adjacency, cluster, directional, percentile, unique) and the minimum number of reads within a group during UMI consensus (1–8, 10, 15). (**A**) Heatmap of the PR-AUCs generated from shearwater-AND. (**B**) Boxplot of the proportion of remaining data when setting the minimum group sizes as 1–8, 10, 15 during UMI consensus after using the grouping method of “directional”, which is the grouping method applied in the pipeline.

As shown in Figure 5A, grouping methods require UMI tags to be identical, i.e., “unique” and “percentile” obtained lower PR-AUCs (0.21) compared with the network-based grouping methods (“adjacency”, “cluster”, and “directional”) of 0.23 when there was no filtering on the family size of the groups (minimum group size = 1) during UMI consensus. The grouping performances of “unique” and “percentile” were improved by increasing the minimum group size to 3 for UMI consensus, where all grouping methods obtained similar PR-AUCs (0.25). In this case, approximately 90% of the data remained after filtering (Figure 5B, Supplementary Figure S3). The largest PR-AUCs of 0.27 were obtained by setting the minimum group size to 5, and more stringent filtering did not further increase the results. When the minimum group size was set as 15, and only about 45% of the data remained (Figure 5B, Supplementary Figure S3), the resulting PR-AUC of the method ‘unique’ dropped to 0.239, while the values of the other methods still maintained around 0.26.

## 3. Discussion

In contrast to previous benchmarking studies [28], our work utilizes real clinical cfDNA data from cancer patients to provide a comprehensive comparison of bioinformatics processing of somatic variant detection in cfDNA using targeted sequencing with UMIs. We developed a pipeline to detect somatic SNVs in cfDNA using deep targeted UMI-seq data. Four different somatic variant calling methods were benchmarked for both mutation detection and sample classification in tumor-informed and tumor-agnostic contexts. Additionally, we compared distinct UMI strategies. Based on our findings, we can offer some bioinformatics recommendations for researchers analyzing high-depth cfDNA sequencing data with UMIs.

Our results on correctly calling tumor mutations from cfDNA show that the deeper sequencing of the PBMC can increase the precision of mutation detection for all variant callers, indicating the efficient removal of false positive calls, such as sequencing errors and CHIP mutations. We can also recommend filtering recurrent mutation calls from the PON from the variant calls. The effect of this is largest on methods—such as DREAMS and VarScan2—that do not filter or downgrade specific variants based on the PON, but doing so did not have a detrimental effect on the other variant callers either.

Before classifying samples as either cases or controls, it is important to consider systematic differences between the handling of the case and control samples that can confound the analysis. Ideally, samples from cases and controls should be randomized and processed identically. In practice, this is unfortunately not always the case because control samples are collected at different sites and times or because controls are shared between different studies. Our results show that differences in the sequencing depth of the cfDNA samples or PBMC samples are important confounders. If a sample has a higher mean depth than the rest, then the probability of seeing a variant with high quality in that sample will also be different. This will create a big problem for analyses that compare the variant qualities between samples. To alleviate this, we recommend downsampling the sequence data so that all samples have a similar depth before performing any analysis that compares the variant qualities of samples.

When classifying whether a sample contains a tumor using prior knowledge of observed tumor mutations, all variant callers achieved a ROC-AUC greater than 0.89. The best-performing method reached a ROC-AUC of 0.984, demonstrating the strong potential for monitoring patient relapse through liquid biopsies when tumor mutations are identified from surgical biopsies. However, without prior tumor information, tumor-agnostic classification achieved a maximum ROC-AUC of 0.808, highlighting that diagnosing cancer using targeted sequencing of cfDNA is a much harder problem and that, currently, targeted cfDNA sequencing is not ready to be used for broad screening programs.

Our comparison of different variant callers shows that shearwater is best at precisely calling variants and making limited false positive calls. Of the two shearwater methods, the AND method is superior for calling lower frequency SNVs as it requires the alternative allele to appear on merely one of the strands, while the OR method requires the allele to appear on both of the strands. The stringent cutoff of the OR algorithm reduces its ability to call low-frequency ctDNA variants but increases the confidence of those variants it can call. Shearwater-AND was also best for sample classification when tumor-informed information was present. However, when no tumor reference was provided, DREAMS-vc showed the best performance.

DREAMS-vc and shearwater are algorithms developed for detecting low-frequency variants, conducting read-wise modeling and position-wise modeling, respectively. Regarding DREAMS-vc, the general sequencing context and read features help construct an error model for each base of the read, and the derived model can theoretically be applied to call variants outside the target regions within the same sample. However, a shortcoming of DREAMS-vc is that position-specific errors cannot be identified; thus, it frequently calls multiple false positives from the same position, which is shown by the initial drop of the precision–recall curve where DREAMS-vc treats the 1% error signal as a variant signal and assigns it a *p*-value of 0 (Figure 2A,B). Shearwater can better deal with position-specific errors, as it estimates an error distribution based on a beta-binomial model for each locus. Compared with the neural network for the error model training of DREAMS-vc, shearwater runs much faster but relies heavily on the quality of the PON data, which should be generated by the exact same protocols as the plasma data and cannot be generalized to other unsequenced regions.

Regarding Mutect2 and VarScan2, which are methods developed for mainly calling tumor mutations rather than low-frequency ctDNA variants, we observe that their performance on such data is limited. Varscan2 was the only tested method that did not utilize PON data or similar control data devoid of tumor signals for position masking or error modeling; thus, the sequencing errors cannot be efficiently removed from those true rare ctDNA SNVs. Additionally, the performance of VarScan2 is significantly affected by the sequencing depth. Therefore, careful consideration is required when using VarScan2 for sample classification. Even though Mutect2 can utilize PON data, this is not used for background error modeling, but merely masks the positions seen in both tumor samples and the PON.

In addition to variant caller benchmarking, we also compared different options for UMI grouping by UMI-tools and the minimum group size for UMI consensus read creation by the fgbio software suite. Sequencing errors can occur not only in the sequence reads but also within UMI tags, so UMI-tools offer various grouping methods to address these UMI errors. Network-based UMI grouping methods, which can merge reads with minor UMI base differences by identifying clusters of closely related UMIs, demonstrated greater robustness against sequencing errors compared to methods like ‘unique’ and ‘percentile,’ which require the exact matching of UMI tags. Our findings suggest that network-based grouping methods perform better than exact-match methods when all data are retained. However, the performance gap decreases when filtering out groups with only one or two reads before generating consensus reads. Therefore, when selecting a minimum group size, it is essential to consider the proportion of data retained for analysis. Our study has certain limitations. Firstly, although the four selected variant callers are commonly used tools for calling tumor variants or detecting low-frequency variants, there are still many other potentially effective methods awaiting comparison. Additionally, despite the robust sample size of 111 and a substantial number of candidate SNVs, the use of more datasets prepared under different protocols would help ensure the generality of the conclusions. Finally, the testing of other informative metrics for evaluating sample classification could possibly impact the final rankings of the variant callers.

In conclusion, our comparison of different variant callers on deep target data shows that the choice of variant caller can depend on the purpose of the study. If the goal is to correctly classify individuals with residual cancer in a tumor-informed setting, then shearwater performed best, but for the tumor-agnostic classification of cancer patients, DREAMS-vc performed better. Furthermore, we have shown that it is preferable to not only perform deep sequencing of the cfDNA, but also of the matching normal sample to avoid calling non-cancer somatic mutations. Finally, our comparison of the effect of sequencing depth on variant quality reveals that it is important to sequence all samples at the same depth to avoid this confounding effect on sample classification.

## 4. Materials and Methods

### 4.1. Patients and Sequencing Data

The study consisted of 111 colorectal cancer patient samples collected at Aarhus University Hospital. The cohort included 34, 48, 24, and 5 stage I, II, III, and IV, patients, respectively. Molecular barcode-tagged, deep targeted fixed panel sequencing (UMI length = 9) was performed on cfDNA from 8 mL plasma samples by Illumina NovaSeq at Aarhus University Hospital, as previously described (PMID: 38673836). The capture panel covered 15,396 bp from regions in 12 genes recurrently mutated in colorectal cancer (Supplementary Table S1). For the cfDNA patient samples, redundant sequencing resulted in an average unadjusted depth of 322,840X (sd = 167,245), and after consensus collapsing of all UMI-families at a mean unique depth of 11,567X (sd = 9533).

For each patient, tumor and normal DNA (from the PBMC) were whole-exome sequenced to average depths of 126X (sd = 91) and 78X (sd = 38), respectively. The normal DNA sequencing data were used to filter non-cancer mutations and SNPs (seen in both cfDNA and PBMC) from somatic variants (i.e., seen only in the tumor DNA and/or the cfDNA). In addition to whole-exome sequencing, molecular barcode-tagged, deep targeted fixed panel sequencing was also performed for the PBMC samples (mean = 374,915X, sd = 94,996). The resulting mean unique depth after UMI-family collapsing was 10,936X (sd = 5050) per sample. A schematic overview of the available patients/samples and their sequencing can be seen in Figure 1B, with detailed information for all patients/samples provided in Supplementary Table S2.

### 4.2. Healthy Controls and Sequencing Data

In total, 46 healthy controls were used as Panel of Normals (PON) for either masking positions seen in both cfDNA samples and PON (Mutect2) or modeling sequencing error rates (shearwater and DREAMS-vc). The cfDNA PON samples with a raw mean depth of 313,714X (sd = 156,063) were processed exactly as the cfDNA patient samples. After UMI-family collapsing, the mean unique depth of the PON samples was 9673X (sd = 5272).

Additionally, another independent cohort of 37 healthy controls was included for sample-level classification analysis. Both plasma (unique mean depth 178,731X, sd = 97,981) and PBMC (unique mean depth 374,915X, sd = 92,996) were processed by molecular barcode-tagged, deep targeted fixed panel sequencing. After UMI-family collapsing, the mean unique depth of the plasma and PBMC data was 8766X (sd = 6054) and 3710X (sd = 916), respectively. Detailed information for all controls/samples can be seen in Supplementary Table S2.

### 4.3. Processing the Data

#### 4.3.1. Calling Tumor Mutations in Tumor Biopsies

Sequence reads from the tumor samples were aligned to hg38 with bwa-mem. Mutect2 [20] (v2.2, GATK4 = 4.3.0) was then used to call tumor mutations under the tumor-normal mode, and the called mutations were subsequently filtered by running LearnReadOrientationModel, CalculateContamination, and FilterMutectCalls. In total, 291 SNV mutations were identified in the 111 tumor samples.

#### 4.3.2. Calling cfDNA Mutations

The bioinformatical pipeline consisted of six main steps: alignment, UMI grouping, consensus read creation, data preprocessing, variant calling, and filtering (Figure 1C). The python-based workflow tool gwf (v2.0.2) [29] was used to implement the pipeline so that all steps could be started with a single command. The pipeline is available at https://github.com/BesenbacherLab/UMIseq_variant_calling (accessed on 30 September 2024).

Paired-end FASTQ data for each sample were aligned to the human genome reference hg38 by BWA-mem [30] (v0.7.17), and all non-primary alignments were discarded. The reads tagged by the connected UMIs within a network under the edit distance of 1 were grouped by UMI-tools [18] (v1.0.1) using the method of ‘directional’. The corrected UMI tag and unique group ID tag from one read was copied to the other read, followed by the regrouping of fgbio [26] (v2.1.0) GroupReadsByUMi using the identical corrected UMI information. The consensus read from the group was called by fgbio CallMolecularConsensusReads, and filtered by fgbio FilterConsensusReads under a minimum group size of 3. The resulting BAM file was converted to FASTQ and remapped to the reference genome, while the UMI information was simultaneously saved using MergeBamAlignment from GATK(GATK4, v4.3.0). Before variant calling, the overlapping reads were soft-clipped and masked with Ns by fgbio ClipBam to avoid double counting of the same variant. The target region was extracted by SAMtools [31] (v1.6) to output the alignments overlapping with the custom BED file. After the fixation of mate-related flags, the obtained BAM file was summarized into a pileup file containing per-base information compared to the reference by pysamstats (v1.1.2).

For variant caller benchmarking, four state-of-the-art variant callers were selected: shearwater [21] (v1.47.0), Mutect2 [20] (v2.2, GATK4 = 4.3.0), VarScan2 [19] (v2.4.6), and DREAMS-vc [22] (v0.0.0.9). Both the AND and OR algorithms of shearwater were tested. Mutect2 and DREAMS-vc took BAM files as input, while shearwater and VarScan2 took pileup and mpileup files as input. The model training of DREAMS-vc extracts the following sequencing context features: trinucleotide context, GC content, and read features: the strand aligned to, number of deletions in the read, the fragment size, UMI group size, UMI mismatches compared with the consensus read, the number of errors around the base, the position of the base in the read, the reference base, and the order of the read being sequenced. To gain a comprehensive understanding of the variant calling ability of each method, the benchmarking dataset of cfDNA are all possible SNV variants at all panel positions where at least one alternative allele is seen in the plasma, instead of focusing solely on the mutations obtained from tumor samples. Since VarScan2 only provides an output for statistically significant SNVs, a *p*-value of 1 was manually assigned to all SNVs missing in the output.

### 4.4. Variant Caller Benchmarking

Mutect2 and VarScan2 used the BAM/mpileup files from normal samples to filter out germline mutations, while the other callers did not. To ensure consistency, we applied a post-calling filtering standard across all variant callers as below. To determine if the deeper sequencing of PBMC samples could improve the filtering of non-cancer mutations and SNPs, we implemented different filtering strategies for the mutations identified in the previous steps. For WES-seq PBMC, the variants with a coverage of less than 10 or a VAF larger than 10% in the PBMC were removed. For deep targeted UMI-seq PBMC, the variants with a coverage of less than 1000 or with a VAF larger than 0.1% in the PBMC were removed. Additional filtering was tested by removing the mismatch positions with a VAF larger than 1% given more than one sample in the PON. To visualize the ability of variant calling, the precision–recall curve (PR-curve) and the area under the curve (PR-AUC) were plotted and calculated by an R package yardstick [32] (v1.2.0).

To further assess the mutation detection abilities of the variant callers, we plotted the precision–recall (PR) curves stratified by variant allele frequency (VAF) ranges in plasma: (0, 1 × 10^−4^], (1 × 10^−4^, 1 × 10^−3^], (1 × 10^−3^, 1 × 10^−2^], and (1 × 10^−2^, 1]. The VAF of the cfDNA variants was calculated by dividing the number of alternative (ALT) reads by the total depth of coverage at each position in the cfDNA samples. We calculated the area under the curve (AUC) for each sub-panel and included these values in the figure for comparison. For sample classification under either a tumor-informed or tumor-agnostic setting, deep targeted UMI-seq PBMC samples were selected for filtering non-cancer mutations and SNPs for the cases. Filtering by recurrent mismatch positions in PON was also performed for both cases and controls. To visualize the ability of sample classification, the ROC curve and its area under the curve (ROC-AUC) was plotted and calculated by R package pROC [33] (v1.18.4).

Specifically, for tumor-informed sample classification, each of the 37 control samples was randomly paired with three case samples, thus creating 111 case–control pairs. For each control sample, its tumor-informed mutations were derived from the paired case sample. We evaluated two criteria: the combined statistic of tumor-informed mutations in cfDNA, and the statistic of the highest-ranking tumor-informed mutation in cfDNA, comparing cases and controls. Here, the statistic refers to the TLOD for Mutect2, *p*-value for VarScan2 and DREAMS-vc, and posterior probability for shearwater (assuming a prior probability of 0.5, the posterior probability becomes $\frac{Bayes\;factor}{1+Bayes\;factor}$), respectively. To combine the statistics of multiple tumor-informed variants, we averaged the TLOD values for Mutect2, multiplied the Bayes factors for shearwater, and applied Fisher’s method to the *p*-values for DREAMS-vc and VarScan2. In addition, the highest-ranking variant refers to the variant with the highest TLOD, lowest *p*-value, or lowest posterior probability regarding different variant callers. Therefore, the hypotheses were that the combined statistic of all tumor-informed variants in cfDNA in cases was more significant compared with the simulated tumor-informed variants in cfDNA in controls; and the statistic of the highest-ranking tumor-informed variant in cfDNA in cases was more significant compared with the simulated highest-ranking tumor-informed variant in cfDNA in controls.

For tumor-agnostic sample classification, three different criteria were used for evaluation. The false positive rate (FPR) cutoff was determined by combining all variants that passed the filters from both cases and controls, and then counting the number of false positives (FPs) by subtracting the true positives (tumor-informed variants in cases) from the total number. The cutoff was set using the statistic of the variant ranked at the FP × 0.05% position. On average, this method would result in ~2.2 variant calls per sample. The first evaluation compared the number of called mutations with an FPR cutoff of 0.05%. The second evaluation compared the mean variant allele frequency (VAF) of called variants at a 0.05% FPR cutoff. The final evaluation compared the statistic of the highest-ranking mutation from each sample between cases and controls. Therefore, the hypotheses were that the number of called variants at a 0.05% FPR cutoff in cfDNA in cases was larger compared with that in controls; the mean VAF of called variants at a 0.05% FPR cutoff in cfDNA in cases was higher compared with that in controls; and the statistic of the highest-ranking variant in cfDNA in cases was more significant compared with that in controls.

When testing if sequencing depth can be a confounder in sample classification, we defined ‘variant quality’ as $-log_{10}(p-value)$ for VarSacn2 and DREAMS-vc, $-log_{10}(posterior\;probability)$ for shearwater, and TLOD for Mutect2. Therefore, the mutation with a higher variant quality has a greater possibility to be called.

## Data Availability

The sequencing data analyzed in this study are the same data that were previously used in Frydendahl et al. 2024 [34]. Due to privacy restrictions, it cannot be made publicly available, but it is available through controlled access from GenomeDK (https://genome.au.dk/library/GDK000009). The pipeline used to perform all steps from UMI handling to variant calling is publicly available at https://github.com/BesenbacherLab/UMIseq_variant_calling (accessed on 30 September 2024).

## Supplementary Materials

The following supporting information can be downloaded at: https://www.mdpi.com/article/10.3390/ijms252111439/s1.
