# Supplementary material for: Evaluating Bioinformatics Processing of Somatic Variant Detection in cfDNA Using Targeted Sequencing with UMIs
Source: Int J Mol Sci. 2024 Oct 24;25(21):11439. doi: 10.3390/ijms252111439 (PMC11546253; doi:10.3390/ijms252111439)
Supplement: Supplementary file 1 [file ijms-25-11439-s001.zip › ijms-3263462-supplementary.pdf]

Supplementary Materials

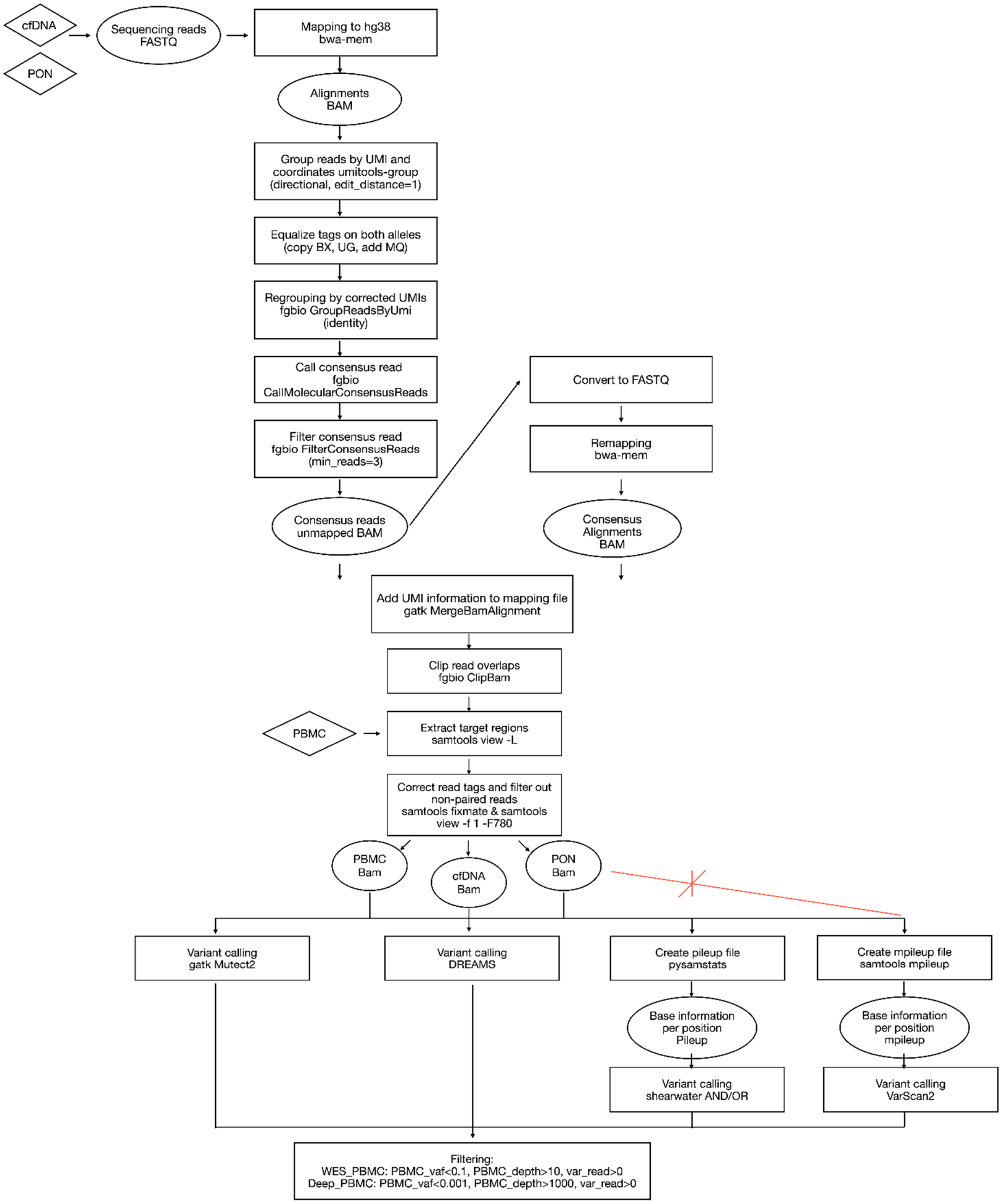

Supp. Figure S1. The detailed flowchart of the pipeline.

Supp. Table S1. Information of the 12-gene target panel.

Gene	Chromosome	Start	End
NRAS	chr1	114713777	114714014
NRAS	chr1	114715992	114716239
PIK3CA	chr3	179198931	179199247
PIK3CA	chr3	179203626	179203917
PIK3CA	chr3	179218166	179218433
PIK3CA	chr3	179234161	179234430
FBXW7	chr4	152324113	152324436
FBXW7	chr4	152325968	152326244
FBXW7	chr4	152326273	152326338
FBXW7	chr4	152328108	152328446
APC	chr5	112780699	112781011
APC	chr5	112792319	112792644
APC	chr5	112801214	112801414
APC	chr5	112815384	112815658
APC	chr5	112819104	112819434
APC	chr5	112826999	112827316
APC	chr5	112827829	112828031
APC	chr5	112828754	112829081
APC	chr5	112834864	112835108
APC	chr5	112837446	112840443
APC	chr5	112840744	112840984
BRAF	chr7	140753212	140753464
TCF7L2	chr10	113141129	113141434
TCF7L2	chr10	113152254	113152542
TCF7L2	chr10	113157894	113158137
TCF7L2	chr10	113165429	113165705

KRAS	chr12	25225504	25225843
KRAS	chr12	25227184	25227467
KRAS	chr12	25245199	25245483
TP53	chr17	7670551	7670845
TP53	chr17	7673406	7673927
TP53	chr17	7674056	7674351
TP53	chr17	7674761	7675365
TP53	chr17	7675871	7676194
SOX9	chr17	72123467	72124262
SMAD2	chr18	47841718	47841958
SMAD2	chr18	47848378	47848698
SMAD4	chr18	51065384	51065673
SMAD4	chr18	51078209	51078516
FAM123B	chrX	64191239	64191626
FAM123B	chrX	64191954	64192094
FAM123B	chrX	64192139	64192353

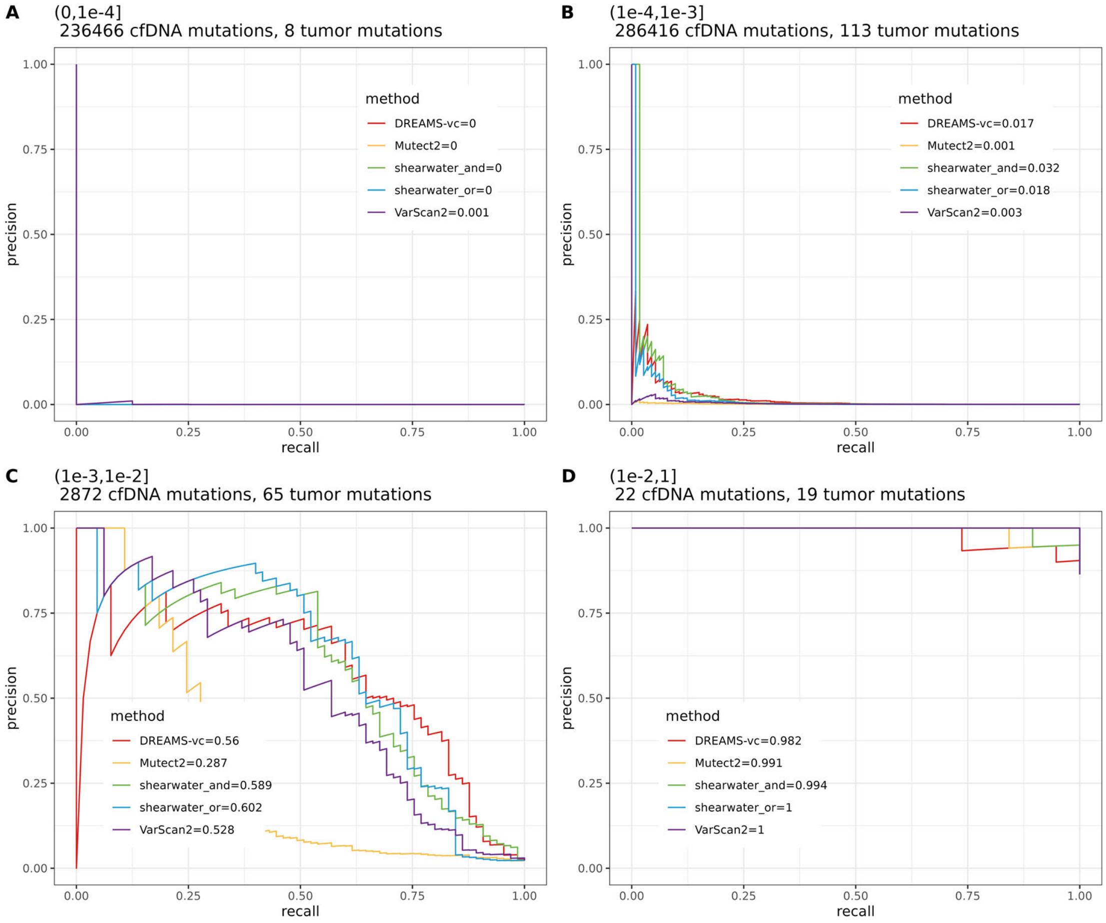

Supp. Figure S2. PR-curve for mutation classification stratified by cfDNA mutation VAF ranges, when using deep targeted UMI-seq PBMC to filter non-tumor mutations, with additional filtering of the problematic regions indicated by PON, which have larger than 1% mismatch rate and are detected in more than one normal sample. **A.** cfDNA mutations with VAF<=1×10^{-4}. Among 236466 cfDNA mutations, 8 mutations are from validation sets (tumor). **B.** cfDNA mutations with 1×10^{-4}<VAF<=1×10^{-3}. Among 286416 cfDNA mutations, 113 mutations are from validation sets (tumor). **C.** cfDNA mutations with 1×10^{-3}<VAF<=1×10^{-2}. Among 2872 cfDNA mutations, 65 mutations are from validation sets (tumor). **D.** cfDNA mutations with 1×10^{-2}<VAF<=1. Among 22 cfDNA mutations, 18 mutations are from validation sets (tumor).

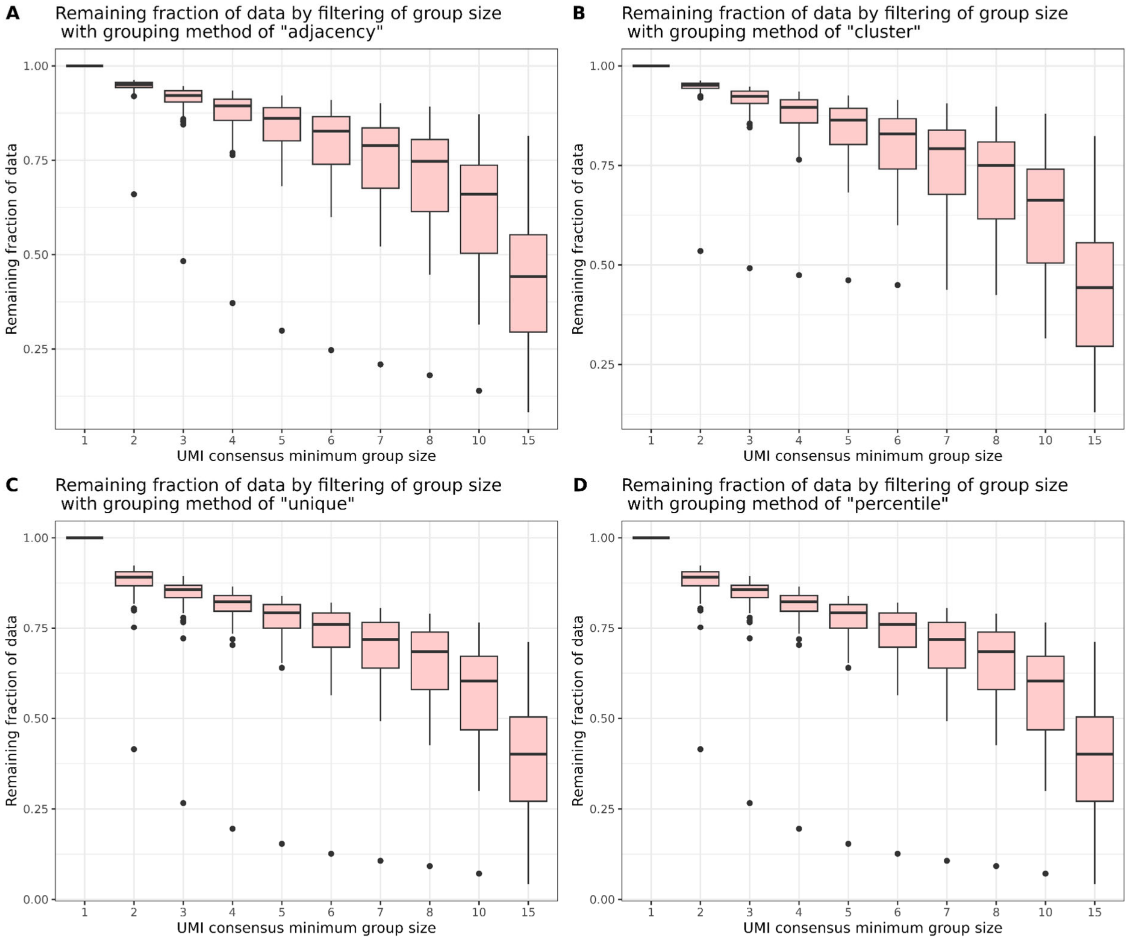

Supp. Figure S3. A. Boxplot of the proportion of remaining data when setting the minimum group sizes as 1-8, 10, 15 during UMI consensus after using the grouping method of "adjacency". **B.** Boxplot of the proportion of remaining data when setting the minimum group sizes as 1-8, 10, 15 during UMI consensus after using the grouping method of "cluster". **C.** Boxplot of the proportion of remaining data when setting the minimum group sizes as 1-8, 10, 15 during UMI consensus after using the grouping method of "unique". **D.** Boxplot of the proportion of remaining data when setting the minimum group sizes as 1-8, 10, 15 during UMI consensus after using the grouping method of "percentile".

Supp. Table S2. Information on the samples used in the study

Type	Sample	Number	Sequencing Strategy	Mean Depth
Case	Plasma	111 (Stage I=34, Stage II=48, Stage III=24, Stage IV=5)	Deep targeted UMI-tagged sequencing	11567X after UMI collapsing
	PBMC	111	Deep targeted UMI-tagged sequencing	10936X after UMI collapsing
			WES	78X
	Tumor	111	WES	126X
Control-I (PON)	Plasma	46	Deep targeted UMI-tagged sequencing	9673X after UMI collapsing
Control-II (Sample Classification)	Plasma	37	Deep targeted UMI-tagged sequencing	8766X after UMI collapsing
	PBMC	37	Deep targeted UMI-tagged sequencing	3710X after UMI collapsing
